# Poly[[(μ-1*H*-benzimidazole-5,6-dicarboxyl­ato)zinc(II)] monohydrate]

**DOI:** 10.1107/S1600536809022107

**Published:** 2009-06-13

**Authors:** Zhao-yang Li, Jing-wei Dai, Shan-tang Yue

**Affiliations:** a School of Chemistry and the Environment, South China Normal University, Guangzhou 510006, People’s Republic of China

## Abstract

The three-dimensional polymeric title compound, {[Zn(C_9_H_4_N_2_O_4_)]·H_2_O}_*n*_, contains one crystallographically independent Zn^II^ atom, one fully deprotonated 1*H*-benzimid­azole-5,6-dicarboxyl­ate (bdc) ligand and one uncoordinated water mol­ecule. The Zn^II^ atom is four-coordinated by three O atoms and one N atom from the bdc ligands, giving a distorted tetra­hedral coordination geometry. The uncoordinated water mol­ecule is bound to the main structure through a strong bdc–water N—H⋯O hydrogen bond, and two much weaker water–bdc O—H⋯O inter­actions.

## Related literature

For structures of other bdc complexes, see: Gao *et al.* (2008 ▶); Lo *et al.* (2007 ▶); Wei *et al.* (2008 ▶); Yao *et al.* (2008 ▶).
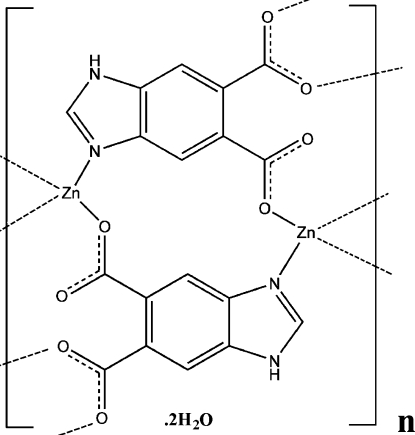


## Experimental

### 

#### Crystal data

[Zn(C_9_H_4_N_2_O_4_)]·H_2_O
*M* *_r_* = 287.55
Monoclinic, 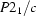

*a* = 6.4735 (5) Å
*b* = 8.1836 (6) Å
*c* = 18.4407 (12) Å
β = 104.397 (2)°
*V* = 946.25 (12) Å^3^
*Z* = 4
Mo *K*α radiation
μ = 2.61 mm^−1^
*T* = 298 K
0.35 × 0.26 × 0.18 mm

#### Data collection

Bruker APEXII area-detector diffractometer
Absorption correction: multi-scan (*SADABS*; Sheldrick, 2004 ▶) *T*_min_ = 0.444, *T*_max_ = 0.625
4613 measured reflections
1665 independent reflections
1513 reflections with *I* > 2σ(*I*)
*R*_int_ = 0.019

#### Refinement

*R*[*F* ^2^ > 2σ(*F* ^2^)] = 0.026
*wR*(*F* ^2^) = 0.072
*S* = 1.06
1665 reflections
154 parameters
H-atom parameters constrained
Δρ_max_ = 0.54 e Å^−3^
Δρ_min_ = −0.32 e Å^−3^

### 

Data collection: *APEX2* (Bruker, 2004 ▶); cell refinement: *SAINT* (Bruker, 2004 ▶); data reduction: *SAINT*; program(s) used to solve structure: *SHELXS97* (Sheldrick, 2008 ▶); program(s) used to refine structure: *SHELXL97* (Sheldrick, 2008 ▶); molecular graphics: *XP* in *SHELXTL* (Sheldrick, 2008 ▶); software used to prepare material for publication: *SHELXL97*.

## Supplementary Material

Crystal structure: contains datablocks I, global. DOI: 10.1107/S1600536809022107/bg2261sup1.cif

Structure factors: contains datablocks I. DOI: 10.1107/S1600536809022107/bg2261Isup2.hkl

Additional supplementary materials:  crystallographic information; 3D view; checkCIF report

## Figures and Tables

**Table 1:** Hydrogen-bond geometry (Å, °)

*D*—H⋯*A*	*D*—H	H⋯*A*	*D*⋯*A*	*D*—H⋯*A*
N2—H2⋯O1*W*	0.86	2.02	2.809 (3)	152
O1*W*—H1*W*⋯O1^i^	0.93	2.45	3.211 (5)	139
O1*W*—H2*W*⋯O4^ii^	0.91	2.29	3.095 (3)	146

Symmetry codes: (i) 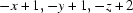; (ii) 
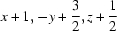
.

**Fig. 1.**
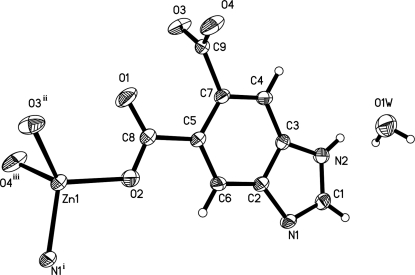
Displacement ellipsoid plot (50% probability level) of the title compound, with atom numbering. Symmetry codes: (i) -x, -y+2, -z+2; (ii) -x, y+1/2, -z+3/2; (iii) x-1, y, z.

**Fig. 2.**
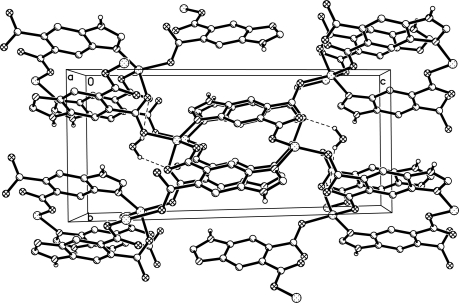
The packing diagram of the title compound, with H atoms omitted for clarity. Hydrogen bonds are shown as dashed lines.

## supplementary crystallographic information

### Comment

N-Heterocyclic carboxylic acids have atracted attention not only because their versatile coordination modes but also owing to their forming high-dimensional polymers through H-bonding interactions. 1*H*-benzimidazole-5,6-dicarboxylic acid (bdc), having six coordination points, is a good candidate for the generation of three-dimensional coordination polymers. However, up to now the complexes based on the bdc ligand are rare (Lo *et al.*, 2007; Gao *et al.*, 2008; Wei *et al.*, 2008; Yao *et al.*, 2008). Here we report the first three-dimensional Zn coordination polymer connected by bdc ligands, [Zn(C_9_H_4_N_2_O_2_)].H_2_O.

As is shown in Figure 1, the asymmetric unit consists of one Zn^2+^ cation, a fully deprotonated bdc^2-^ ligand, and a free water moelcule. The cation has a tetrahedral coordination environment, and is surrounded by three oxygen and one nitrogen atoms from the bdc ligands. A packing diagram showing the 3D structure coming out from the tetradentate character of the bdc ligand is shown in Figure 2. To the best of our knowledge, the title compound is the first 3D transition metal coordination polymer based on the 1*H*-benzimidazole-5,6-dicarboxylic acid ligand.

### Experimental

A mixture of bdc (0.0415 g, 0.20 mmol), Zn(NO_3_)_2_.6H_2_O (0.0594 g, 0.20 mmol) and water (10 ml) was heated up to 430 K for 72 h in a 23 ml Teflon-lined stainless-steel autoclave and then cooled down to room temperature in a 278 K/hour rate. Colourless prismatic crystals were collected and dried in air.

### Refinement

H atoms attached to carbon and nitrogen were placed at calculated positions and treated as riding on their parent atoms with C—H = 0.93Å, C—N = 0.86Å.  Those attached to oxygen  were found from the Fourier maps, and allowed to ride without further refinement. In all cases *U*ĩso~(H) = 1.2 or 1.5 *U*~eq~(C, O).

### Crystal data

**Table:**

[Zn(C_9_H_4_N_2_O_4_)]·H_2_O	*F*(000) = 576
*M**_r_* = 287.55	*D*_x_ = 2.018 Mg m^−^^3^
Monoclinic, *P*2_1_/*c*	Mo *K*α radiation, λ = 0.71073 Å
Hall symbol: -P 2ybc	Cell parameters from 2639 reflections
*a* = 6.4735 (5) Å	θ = 2.3–27.6°
*b* = 8.1836 (6) Å	µ = 2.61 mm^−^^1^
*c* = 18.4407 (12) Å	*T* = 298 K
β = 104.397 (2)°	Block, colorless
*V* = 946.25 (12) Å^3^	0.35 × 0.26 × 0.18 mm
*Z* = 4	

### Data collection

**Table:**

Bruker APEXII area-detector diffractometer	1665 independent reflections
Radiation source: fine-focus sealed tube	1513 reflections with *I* > 2σ(*I*)
graphite	*R*_int_ = 0.019
φ and ω scans	θ_max_ = 25.0°, θ_min_ = 2.3°
Absorption correction: multi-scan (*SADABS*; Sheldrick, 2004)	*h* = −7→7
*T*_min_ = 0.444, *T*_max_ = 0.625	*k* = −9→8
4613 measured reflections	*l* = −21→16

### Refinement

**Table:**

Refinement on *F*^2^	Primary atom site location: structure-invariant direct methods
Least-squares matrix: full	Secondary atom site location: difference Fourier map
*R*[*F*^2^ > 2σ(*F*^2^)] = 0.026	Hydrogen site location: inferred from neighbouring sites
*wR*(*F*^2^) = 0.072	H-atom parameters constrained
*S* = 1.06	*w* = 1/[σ^2^(*F*_o_^2^) + (0.0407*P*)^2^ + 0.8076*P*] where *P* = (*F*_o_^2^ + 2*F*_c_^2^)/3
1665 reflections	(Δ/σ)_max_ = 0.001
154 parameters	Δρ_max_ = 0.54 e Å^−^^3^
0 restraints	Δρ_min_ = −0.32 e Å^−^^3^

### Special details

**Table:**

Geometry. All e.s.d.'s (except the e.s.d. in the dihedral angle between two l.s. planes) are estimated using the full covariance matrix. The cell e.s.d.'s are taken into account individually in the estimation of e.s.d.'s in distances, angles and torsion angles; correlations between e.s.d.'s in cell parameters are only used when they are defined by crystal symmetry. An approximate (isotropic) treatment of cell e.s.d.'s is used for estimating e.s.d.'s involving l.s. planes.
Refinement. Refinement of *F*^2^ against ALL reflections. The weighted *R*-factor *wR* and goodness of fit *S* are based on *F*^2^, conventional *R*-factors *R* are based on *F*, with *F* set to zero for negative *F*^2^. The threshold expression of *F*^2^ > σ(*F*^2^) is used only for calculating *R*-factors(gt) *etc*. and is not relevant to the choice of reflections for refinement. *R*-factors based on *F*^2^ are statistically about twice as large as those based on *F*, and *R*- factors based on ALL data will be even larger.

### Fractional atomic coordinates and isotropic or equivalent isotropic displacement parameters (Å^2^)

**Table:**

	*x*	*y*	*z*	*U*_iso_*/*U*_eq_	
C1	0.6805 (4)	0.7618 (3)	1.14674 (15)	0.0244 (6)	
H1	0.7562	0.7565	1.1967	0.029*	
C2	0.4362 (4)	0.8133 (3)	1.04587 (14)	0.0210 (6)	
C3	0.5993 (4)	0.7274 (3)	1.02485 (14)	0.0209 (6)	
C4	0.5896 (4)	0.6922 (3)	0.95017 (14)	0.0232 (6)	
H4	0.6986	0.6358	0.9364	0.028*	
C5	0.2433 (4)	0.8286 (3)	0.91858 (14)	0.0210 (6)	
C6	0.2567 (4)	0.8630 (3)	0.99290 (14)	0.0225 (6)	
H6	0.1474	0.9184	1.0070	0.027*	
C7	0.4108 (4)	0.7447 (3)	0.89738 (14)	0.0198 (5)	
C8	0.0478 (4)	0.8790 (3)	0.85989 (15)	0.0244 (6)	
C9	0.4056 (4)	0.7163 (3)	0.81632 (14)	0.0200 (6)	
N1	0.4937 (3)	0.8337 (3)	1.12397 (12)	0.0223 (5)	
N2	0.7500 (3)	0.6973 (3)	1.09061 (12)	0.0255 (5)	
H2	0.8682	0.6459	1.0948	0.031*	
O1	0.0221 (4)	0.8307 (4)	0.79625 (13)	0.0700 (10)	
O2	−0.0815 (3)	0.9719 (3)	0.88154 (11)	0.0371 (5)	
O3	0.3461 (3)	0.5800 (3)	0.78853 (10)	0.0317 (5)	
O4	0.4720 (4)	0.8254 (3)	0.78041 (11)	0.0360 (5)	
Zn1	−0.35253 (5)	1.02462 (4)	0.814219 (16)	0.02108 (13)	
O1W	1.1216 (4)	0.5187 (3)	1.15400 (15)	0.0533 (7)	
H1W	1.0604	0.4556	1.1853	0.080*	
H2W	1.1968	0.6024	1.1810	0.080*	

### Atomic displacement parameters (Å^2^)

**Table:**

	*U*^11^	*U*^22^	*U*^33^	*U*^12^	*U*^13^	*U*^23^
C1	0.0238 (14)	0.0330 (16)	0.0141 (13)	0.0009 (12)	0.0004 (11)	0.0029 (11)
C2	0.0226 (13)	0.0263 (14)	0.0138 (13)	0.0008 (11)	0.0037 (11)	−0.0001 (10)
C3	0.0189 (13)	0.0270 (15)	0.0153 (13)	0.0023 (11)	0.0013 (11)	0.0010 (11)
C4	0.0215 (13)	0.0306 (15)	0.0177 (14)	0.0056 (11)	0.0053 (11)	−0.0025 (11)
C5	0.0212 (13)	0.0264 (15)	0.0146 (13)	0.0006 (11)	0.0029 (11)	−0.0019 (10)
C6	0.0206 (13)	0.0310 (15)	0.0159 (13)	0.0052 (11)	0.0046 (11)	−0.0016 (11)
C7	0.0222 (13)	0.0220 (14)	0.0151 (13)	−0.0006 (11)	0.0043 (10)	−0.0015 (10)
C8	0.0230 (14)	0.0329 (16)	0.0160 (14)	0.0016 (12)	0.0025 (11)	−0.0013 (11)
C9	0.0174 (12)	0.0266 (15)	0.0152 (12)	0.0018 (10)	0.0025 (10)	−0.0009 (11)
N1	0.0239 (12)	0.0313 (13)	0.0107 (11)	0.0047 (10)	0.0026 (9)	0.0001 (9)
N2	0.0208 (12)	0.0352 (14)	0.0189 (12)	0.0083 (10)	0.0017 (9)	0.0002 (10)
O1	0.0496 (15)	0.125 (3)	0.0230 (13)	0.0460 (16)	−0.0143 (11)	−0.0294 (14)
O2	0.0285 (11)	0.0578 (15)	0.0209 (11)	0.0200 (10)	−0.0012 (9)	−0.0072 (9)
O3	0.0511 (13)	0.0269 (11)	0.0190 (10)	−0.0077 (10)	0.0125 (9)	−0.0057 (8)
O4	0.0532 (14)	0.0376 (13)	0.0185 (10)	−0.0200 (10)	0.0117 (10)	−0.0050 (9)
Zn1	0.0221 (2)	0.0270 (2)	0.01325 (19)	0.00140 (12)	0.00268 (13)	−0.00017 (12)
O1W	0.0410 (14)	0.0652 (18)	0.0468 (16)	0.0148 (12)	−0.0022 (12)	−0.0085 (12)

### Geometric parameters (Å, °)

**Table:**

C1—N1	1.316 (3)	C6—H6	0.9300
C1—N2	1.336 (3)	C7—C9	1.505 (4)
C1—H1	0.9300	C8—O1	1.210 (3)
C2—C6	1.380 (4)	C8—O2	1.266 (3)
C2—C3	1.401 (4)	C9—O3	1.247 (3)
C2—N1	1.405 (3)	C9—O4	1.250 (3)
C3—N2	1.376 (3)	N2—H2	0.8600
C3—C4	1.393 (4)	Zn1—O2	1.929 (2)
C4—C7	1.383 (4)	Zn1—N1^i^	1.999 (2)
C4—H4	0.9300	Zn1—O3^ii^	1.9587 (19)
C5—C6	1.381 (4)	Zn1—O4^iii^	1.996 (2)
C5—C7	1.418 (4)	O1W—H1W	0.9333
C5—C8	1.504 (4)	O1W—H2W	0.9127
			
N1—C1—N2	112.9 (2)	O1—C8—C5	119.8 (3)
N1—C1—H1	123.6	O2—C8—C5	116.2 (2)
N2—C1—H1	123.6	O3—C9—O4	122.2 (2)
C6—C2—C3	120.8 (2)	O3—C9—C7	118.3 (2)
C6—C2—N1	130.8 (2)	O4—C9—C7	119.3 (2)
C3—C2—N1	108.5 (2)	C1—N1—C2	105.2 (2)
N2—C3—C4	133.0 (2)	C1—N1—Zn1^i^	126.26 (18)
N2—C3—C2	105.3 (2)	C2—N1—Zn1^i^	127.64 (17)
C4—C3—C2	121.7 (2)	C1—N2—C3	108.1 (2)
C7—C4—C3	117.1 (2)	C1—N2—H2	125.9
C7—C4—H4	121.4	C3—N2—H2	125.9
C3—C4—H4	121.4	C8—O2—Zn1	119.85 (18)
C6—C5—C7	120.6 (2)	C9—O3—Zn1^iv^	121.73 (18)
C6—C5—C8	119.5 (2)	C9—O4—Zn1^v^	131.46 (18)
C7—C5—C8	119.9 (2)	O2—Zn1—O3^ii^	116.08 (9)
C2—C6—C5	118.5 (2)	O2—Zn1—O4^iii^	111.99 (10)
C2—C6—H6	120.8	O3^ii^—Zn1—O4^iii^	91.96 (9)
C5—C6—H6	120.8	O2—Zn1—N1^i^	103.56 (9)
C4—C7—C5	121.3 (2)	O3^ii^—Zn1—N1^i^	122.67 (9)
C4—C7—C9	117.4 (2)	O4^iii^—Zn1—N1^i^	110.27 (9)
C5—C7—C9	121.3 (2)	H1W—O1W—H2W	109.2
O1—C8—O2	124.0 (3)		
			
C6—C2—C3—N2	−179.7 (3)	C5—C7—C9—O3	−99.2 (3)
N1—C2—C3—N2	0.4 (3)	C4—C7—C9—O4	−92.4 (3)
C6—C2—C3—C4	1.5 (4)	C5—C7—C9—O4	84.8 (3)
N1—C2—C3—C4	−178.4 (3)	N2—C1—N1—C2	0.3 (3)
N2—C3—C4—C7	−178.8 (3)	N2—C1—N1—Zn1^i^	−169.63 (19)
C2—C3—C4—C7	−0.5 (4)	C6—C2—N1—C1	179.7 (3)
C3—C2—C6—C5	−1.1 (4)	C3—C2—N1—C1	−0.4 (3)
N1—C2—C6—C5	178.8 (3)	C6—C2—N1—Zn1^i^	−10.6 (4)
C7—C5—C6—C2	−0.2 (4)	C3—C2—N1—Zn1^i^	169.32 (18)
C8—C5—C6—C2	179.0 (3)	N1—C1—N2—C3	0.0 (3)
C3—C4—C7—C5	−0.9 (4)	C4—C3—N2—C1	178.4 (3)
C3—C4—C7—C9	176.3 (2)	C2—C3—N2—C1	−0.2 (3)
C6—C5—C7—C4	1.3 (4)	O1—C8—O2—Zn1	7.0 (5)
C8—C5—C7—C4	−178.0 (3)	C5—C8—O2—Zn1	−173.02 (19)
C6—C5—C7—C9	−175.8 (3)	O4—C9—O3—Zn1^iv^	0.2 (4)
C8—C5—C7—C9	4.9 (4)	C7—C9—O3—Zn1^iv^	−175.59 (17)
C6—C5—C8—O1	−170.2 (3)	O3—C9—O4—Zn1^v^	−160.8 (2)
C7—C5—C8—O1	9.1 (5)	C7—C9—O4—Zn1^v^	14.9 (4)
C6—C5—C8—O2	9.8 (4)	C8—O2—Zn1—O3^ii^	−44.1 (3)
C7—C5—C8—O2	−170.9 (3)	C8—O2—Zn1—O4^iii^	59.7 (2)
C4—C7—C9—O3	83.6 (3)	C8—O2—Zn1—N1^i^	178.5 (2)

Symmetry codes: (i) −*x*, −*y*+2, −*z*+2; (ii) −*x*, *y*+1/2, −*z*+3/2; (iii) *x*−1, *y*, *z*; (iv) −*x*, *y*−1/2, −*z*+3/2; (v) *x*+1, *y*, *z*.

### Hydrogen-bond geometry (Å, °)

**Table:**

*D*—H···*A*	*D*—H	H···*A*	*D*···*A*	*D*—H···*A*
N2—H2···O1W	0.86	2.02	2.809 (3)	152
O1W—H1W···O1^vi^	0.93	2.45	3.211 (5)	139
O1W—H2W···O4^vii^	0.91	2.29	3.095 (3)	146

Symmetry codes: (vi) −*x*+1, −*y*+1, −*z*+2; (vii) *x*+1, −*y*+3/2, *z*+1/2.
